# Closed-system behaviour of the intra-crystalline fraction of amino acids in mollusc shells

**DOI:** 10.1016/j.quageo.2007.07.001

**Published:** 2008-02

**Authors:** K.E.H. Penkman, D.S. Kaufman, D. Maddy, M.J. Collins

**Affiliations:** a BioArch, Departments of Biology, Archaeology and Chemistry, Biology S Block, University of York, P.O. Box 373, York, YO10 5YW, UK; b Department of Geology, Northern Arizona University, Flagstaff, AZ 86011, USA; c School of Geography, Politics and Sociology, Daysh Building, Newcastle University, Newcastle-upon-Tyne, NE1 7RU, UK

**Keywords:** Dating, Quaternary, Intra-crystalline protein degradation, Racemization

## Abstract

When mollusc shells are analysed conventionally for amino acid geochronology, the entire population of amino acids is included, both inter- and intra-crystalline. This study investigates the utility of removing the amino acids that are most susceptible to environmental effects by isolating the fraction of amino acids encapsulated within mineral crystals of mollusc shells (intra-crystalline fraction). Bleaching, heating and leaching (diffusive loss) experiments were undertaken on modern and fossil *Corbicula fluminalis*, *Margaritifera falcata*, *Bithynia tentaculata* and *Valvata piscinalis* shells. Exposure of powdered mollusc shells to concentrated NaOCl for 48 h effectively reduced the amino acid content of the four taxa to a residual level, assumed to represent the intra-crystalline fraction. When heated in water at 140 °C for 24 h, only 1% of amino acids were leached from the intra-crystalline fraction of modern shells compared with 40% from whole shell. Free amino acids were more effectively retained in the intra-crystalline fraction, comprising 55% (compared with 18%) of the whole shell after 24 h at 140 °C. For fossil gastropods, the inter-shell variability in D/L values for the intra-crystalline fraction of a single-age population was reduced by 50% compared with conventionally analysed shells. In contrast, analysis of the intra-crystalline fraction of *C. fluminalis* does not appear to improve the results for this taxon, possibly due to variability in shell ultrastructure. Nonetheless, the intra-crystalline fraction in gastropods approximates a closed system of amino acids and appears to provide a superior subset of amino acids for geochronological applications.

## 1 Introduction

The study of organic matter within fossil biominerals typically relies on the assumption that the isolated organic fraction is indigenous (e.g., Engel and Macko, 1986; Engel et al., 1994). If undetected, either contamination or loss of original organic matter will confound interpretations of the history of the fossil. The degradation products of the organic matter also need to be retained within the biomineral if diagenetic reactions are used to assess the original properties and burial history of the sample. Ideally therefore, the investigation of fossil organic material requires a “closed system”, whereby the organic material experiences no chemical or physical interaction with the external environment. Collins and Riley (2000) argued that, to confidently interpret amino acid racemization data for geochronology, the system must remain closed from synthesis to analysis. In a closed system, diagenetic reactions of indigenous biomolecules should be predictable, and the original molecules and their degradation products can be used to interpret the burial history of the sample. A closed system buffered by the mineral has the further advantage of a consistent chemical environment (narrow pH, cation distribution, and free water; Sykes et al., 1995). Post-hoc comparison of degradation products in such closed systems can be used to interpret the burial history of the sample.

The success of avian eggshell as a material for amino acid geochronology has been attributed to its approximation to a closed system (Brooks et al., 1990; Miller et al., 2000). Ostrich eggshell heated at 105 °C for 70 h under conditions of continuous leaching (diffusive loss) retained 99% of its original stable amino acid concentration, but under similar laboratory conditions mollusc shell retained only 40% (Miller and Hare, 1980). A substantial proportion of mollusc protein is similarly leached in the geologic environment (Roof, 1997). Nonetheless, Crenshaw (1972) found that a residual fraction of amino acids was retained by mollusc shells, and could be isolated by extensive bleach treatment of shell powders. This protected organic matter comprised between 0.001% and 0.01% by weight of the biomineral. Similar yields following bleaching were reported for brachiopods (Collins et al., 1991), echinoderms (Berman et al., 1993), and foraminifera (Stathoplos and Hare, 1993). The presence in fossil shells of alkyl amines, volatile fatty acids (Hoering, 1980) and light hydrocarbons (Thompson and Creath, 1966), whose molecular characteristics are consistent with an amino acid origin, offer further tantalizing evidence of a trapped organic fraction. Towe and Thompson (1972), using ion beam thinning, observed organic matter between crystallites. This early research led Towe (1980, p. 73) to postulate that “organic material trapped within single crystals during biomineralization offers the best hope for the study of ancient fossil proteins”.

Trapping of organic material within crystals has been reported in previous studies. For example, rhombohedral crystals of calcite incorporated significant amounts of the gel in which they were grown (Nickl and Henisch, 1969; Henisch, 1970). A crystal by definition cannot contain large macromolecules as an integral part of the crystal lattice. Here we adopt the common usage of “intra-crystalline” as an operational term to describe the organic matter fraction resistant to prolonged exposure to strong chemical oxidation (Berman et al., 1993; Sykes et al., 1995; Stern et al., 1999). The precise relationship between the intra-crystalline material and the surrounding mineral is not completely understood, although recent high-resolution SEM and AFM studies identify a common nanoparticulate structure (Dauphin, 2001; Stolarski and Mazur, 2005; Oaki et al., 2006), which is believed to be intimately associated with organic matter—possibly sulphated glycoproteins (Cuif and Dauphin, 2005 and commentaries therein).

Despite the potential superiority of the intra-crystalline fraction for amino acid geochronology, only one investigation has been published on fossil shells (Sykes et al., 1995). Sykes et al. showed that samples with aberrantly young amino acid racemization (AAR) ages determined using conventional preparation procedures produced lower yields of amino acids, but higher and more consistent racemization values after prolonged soaking in sodium hypochlorite (NaOCl). Our study further explores the isolation and diagenetic state of an enclosed proteinaceous component system within mollusc shells. Commonly occurring Quaternary fossil gastropod and bivalve shells of differing size and shell ultra-structure were analysed to assess further the suitability of the bleaching approach (Table 1). Initially, modern shells were subjected to different pre-treatments to optimize the oxidation protocol. The intra-crystalline fraction was then tested at high temperatures to mimic natural diagenesis. Finally fossil shells were also analysed to compare the intra-crystalline fraction with the results from conventional AAR procedures.

## 2 Methods

### 2.1 Pre-treatment procedure

The pre-treatment procedure was optimized during the course of this study (Section 3). Unless stated otherwise, samples were prepared as follows. Individual shells were sonicated and rinsed several times in HPLC-grade water. The shells were air-dried overnight, crushed using a mortar and pestle, and sieved to separate particles between 0.425 and 0.090 mm, then split into two subsamples, unbleached and bleached. For bleached subsamples, approximately 10 mg of powder was transferred to an eppendorf tube and 50 μL of 12% NaOCl (BDH) was added per mg of carbonate. The tubes were shaken, left for 24 h, re-shaken to ensure complete exposure to the bleach, and soaked for a further 24 h. The NaOCl was then pipetted off, the powder rinsed with H_2_O, centrifuged, and rinsed again. This was repeated five times. HPLC-grade Methanol (BDH) was then added to ensure complete removal of bleach, left for a few minutes, centrifuged, and pipetted off. The bleached powder was air dried overnight.

Dry powders (bleached and unbleached) were further split into two subsamples and weighed accurately into sterile glass vials: one for the analysis of the unbound amino acids (free amino acid fraction; FAA or F), and one for all the amino acids present (total hydrolysable amino acids; THAA or H). All samples were prepared using the procedures of Penkman (2005). The FAA subsamples were demineralized with 10 μL 2 M HCl (Aristar) per mg of CaCO_3_ and dried overnight in a centrifugal evaporator. THAA subsamples were demineralized in 20 μL of 7 M HCl (Aristar) per mg of CaCO_3_. The vials were flushed with N_2_ to minimize oxidation reactions, and hydrolysed to release peptide-bound amino acids by heating in a 110 °C oven for 6 h (H6) or 24 h (H*), retightening the caps after 10 min to prevent leakage. The shorter hydrolysis time (6 h) did not appear to break all the peptide bonds, except for those of Asp. This resulted in an increase in concentration of THAA during the initial phases of the heating experiments; therefore in subsequent experiments, 24 h hydrolysis was used. Following hydrolysis, vials were placed in a centrifugal evaporator overnight to dry.

### Analytical procedure

Samples were rehydrated with 0.01 mM HCl containing an internal standard of l-*homo*-arginine, and analysed by reverse-phase high performance liquid chromatography (RP-HPLC) using fluorescence detection following a modified method of Kaufman and Manley (1998). A solution volume of 2 μL was mixed online with 2.2 μL of derivatizing reagent (260 mM *N*-isobutyryl-l-cysteine (IBLC), 170 mM *o*-phthaldialdehyde (OPA) in 1 M potassium borate buffer, adjusted to pH 10.4 with KOH) immediately prior to injection. The derivitized amino acids were separated on a C_18_ HyperSil BDS column (5 mm×250 mm) at 25 °C using a gradient elution of three solvents: sodium acetate buffer (23 mM sodium acetate tri-hydrate, 1.5 mM sodium azide, 1.3 μM EDTA, adjusted to pH 6.00±0.01 with 10% acetic acid and sodium hydroxide), methanol, and acetonitrile. The L and D isomers of 10 amino acids were routinely detected, but the amino acids studied in detail were those whose both D and L enantiomers were well resolved: Asx, Glx, Ser, Ala, Val and Phe. The measure of “Total” concentration includes these amino acids, along with Gly. During hydrolysis, both asparagine and glutamine undergo rapid irreversible deamination to aspartic acid and glutamic acid, respectively (Hill, 1965). Therefore it is not possible to distinguish these amino acids and they are reported as Asx and Glx respectively. Amino acid concentrations were calculated as the mean of the duplicate analyses using peak areas normalized to the internal standard, and expressed as picomoles (pmol) per mg of shell. None of the results were screened. Procedural blanks were included at each step of the preparation process, but levels of amino acids within these blanks were low (<100 pmol/mg “total” content), so no corrections were routinely made. Both intra- and inter-laboratory standards were analysed routinely in order to monitor the performance of the RP-HPLC machines. The values for the inter-laboratory comparison samples (ILC; Wehmiller, 1984; Kaufman and Manley, 1998) are reported in the supplementary information.

## 3 Optimization of oxidation procedure using NaOCl and H_2_O_2_

The previous analyses of Sykes et al. (1995) used strong NaOCl to isolate the intra-crystalline fraction of amino acids. Hydrogen peroxide (H_2_O_2_), a less persistent oxidant, is commonly used in micropalaeontological preparations. Therefore these two oxidants were compared using different size fractions of two species of bivalves: a late Holocene Heterodont *Corbicula fluminalis* (Müller, 1974), and a modern Palaeoheterodont *Margaritifera falcata*. A Holocene fluvial gastropod *Bithynia tentaculata* was also tested using a range of exposure times to NaOCl. These species were chosen because the more primitive *Margaritifera* has a simple shell structure consisting of an inner calcareous nacreous layer and an outer aragonitic prismatic layer. *Corbicula* by contrast is predominately composed of an aragonitic cross-lamellar layer, with a thin prismatic layer. Most gastropods possess a cross-lamellar ultrastructure (Wilmot et al., 1992); in the case of *Bithynia*, the shell is aragonite.

### 3.1 Oxidation experiment procedure

The periostracum was removed from the modern *Margaritifera* shells with a rotary drill. All the shells were cleaned by sonication and rinsed with 18 MΩ water. The bivalve shells were crushed and sieved to three size fractions: coarse=1.000−0.425 mm, medium=0.425−0.090 mm, and fine=0.090−0.045 mm. The *Bithynia* shell was prepared only as the medium size fraction. The powders were then rinsed in 18 MΩ water to remove adhering dust, and air-dried before the oxidation treatments. Approximately 40 mg of powdered shell was weighed accurately into sterile glass test tubes, and 2 mL of either 12% NaOCl or 30% AnalaR grade H_2_O_2_ was added. Two to five replicates were analysed at each time-step. The test tubes were agitated daily to ensure complete exposure to the oxidants. At designated time intervals, the oxidant was removed, the powders rinsed six times with 18 MΩ water, and 1 mL of HPLC-grade MeOH was added as described above.

### 3.2 Oxidation experiment results

#### 3.2.1 Changes in amino acid concentration with oxidation

**Fig. 1:**
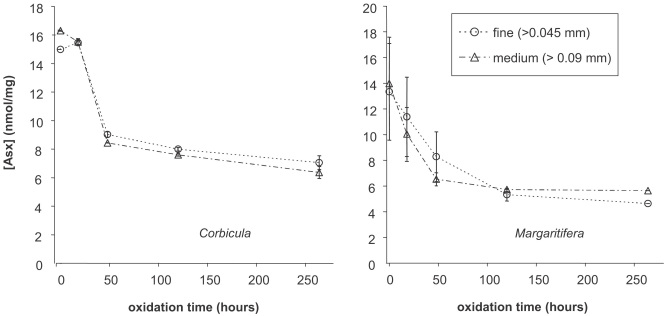
Change in THAA concentration (6 h hydrolysis) of Asx with oxidation time in H_2_O_2_ for *Corbicula* (left) and *Margaritifera* (right).

Although the concentration of THAA decreased with exposure to H_2_O_2_, a stable plateau was not observed over the course of the experiment (Fig. 1). After ten days of oxidation with H_2_O_2_, approximately 50% of the original amino acid concentration remained within the bivalve shell powders.

**Fig. 2:**
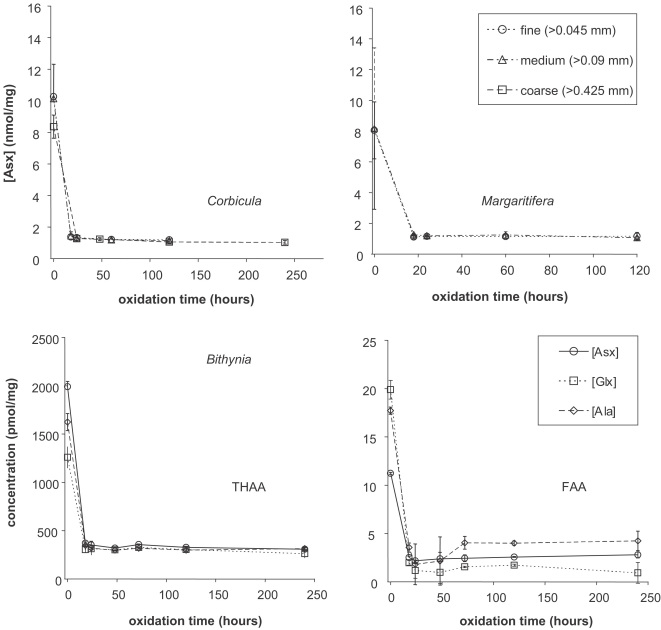
Decline in the amino acid concentration with oxidation time using NaOCl. Upper: THAA concentration of Asx (6 h hydrolysis) for *Corbicula* (left) and *Margaritifera* (right). Other amino acids show similar trends (Fig. 4). The particle size of the powder does not appear to affect the extent of oxidation. Lower: THAA (24 h hydrolysis; left) and FAA (right) concentrations of Asx, Glx, and Ala for *Bithynia.*

**Fig. 3:**
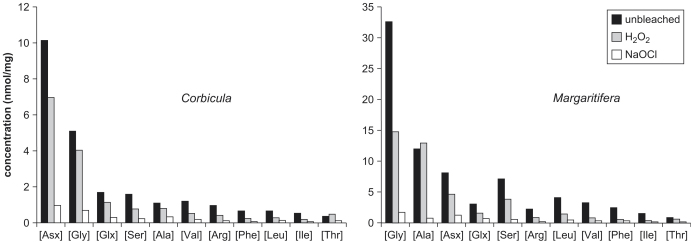
Absolute abundance of 11 amino acids before and after oxidation with H_2_O_2_ and NaOCl for *Corbicula* (left) and *Margaritifera* (right). The concentration of each amino acid decreases on oxidation.

When treated with NaOCl, the concentration of THAA in all three species rapidly decreased. By the first time step (18 h) the concentration of all amino acids was ∼10% of that in the unbleached counterparts (Figs. 2 and 3). On further bleaching, the concentration did not decrease significantly. The particle size of the powder did not affect the rate at which the residual fraction of amino acids was reached, nor the concentration of the residual fraction (Fig. 2, upper). This residual fraction of amino acids measured accounts for approximately 0.005% by weight of mollusc shells, as compared to amino acid content of unbleached shell (0.05%).

The concentration of the FAA also decreased upon bleaching (Fig. 2, lower right). The FAA content within these young samples is low because little protein hydrolysis has occurred. The proportion of FAA increased upon oxidation, representing ∼1.2% of the THAA bleached *Bithynia*, compared with ∼0.6% in the unbleached shell.

#### 3.2.2 Changes in amino acid composition with oxidation

**Fig. 4:**
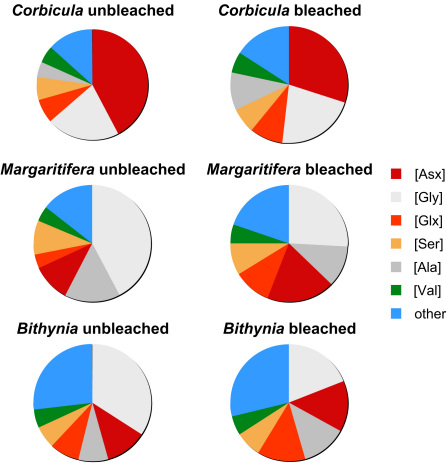
Amino acid composition in *Corbicula*, *Margaritifera* and *Bithynia* shells, unbleached (left) and after oxidation with NaOCl for 48 h (right). The colours for each amino acid follow the convention of RasMol. The concentration of the silk-like protein decreases relative to that of the acidic amino acids in bleached *Margaritifera* and *Bithynia*.

The concentration for all amino acids in the oxidized fraction was higher in the modern shell (*Margaritifera*) than in the Holocene shell (*Corbicula*; Fig. 3). This might reflect species differences in original protein composition, or degradation of amino acids in the Holocene *Corbicula*. The relative proportions of amino acids in the THAA of *Corbicula* remained relatively similar upon oxidation, except for a slight decrease in the proportion of Asx and an increase in the proportion of Ala. Upon oxidation, the relative proportions of Ala and Gly in the THAA of *Margaritifera* decreased significantly, with the Asx and Glx increasing in importance in the bleached fraction. *Bithynia* exhibited a similar pattern of increased acidic amino acids upon bleaching (Fig. 4).

#### 3.2.3 Changes in D/L with NaOCl

**Fig. 5:**
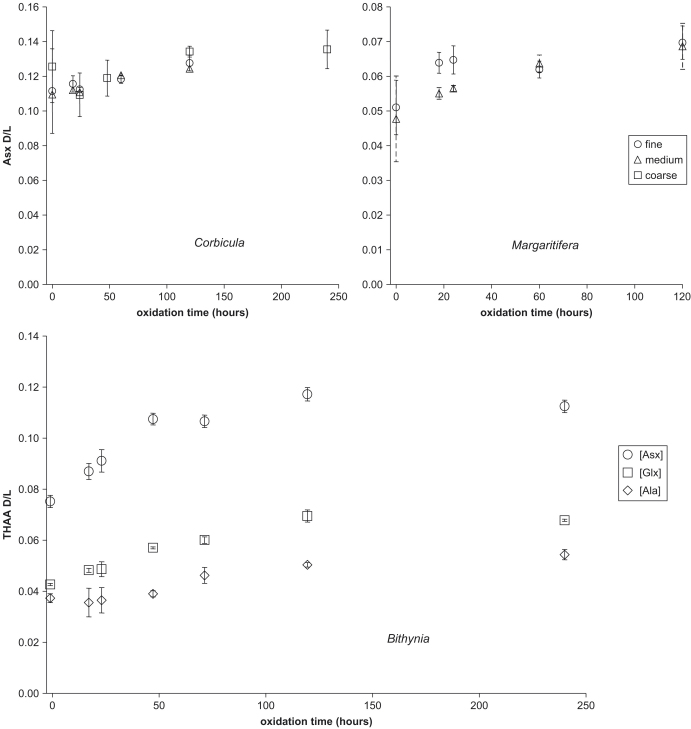
Change in Asx D/L in the THAA with oxidation time using NaOCl. Upper: Asx D/L for *Corbicula* (left) and *Margaritifera* (right). Lower: D/L in Asx, Glx and Ala for *Bithynia.*

Although no significant concentration change was observed subsequent to the first time-step of bleaching, the D/L value of the THAA did increase with increasing oxidation time, for all three species (Fig. 5). The D/L value increased rapidly between the first two time steps, and then levelled off with increasing bleaching time, but a plateau was not reached within the period analysed.

### 3.3 Discussion of oxidation experiments

Oxidation using H_2_O_2_ was less effective at decomposing amino acids than NaOCl (Figs. 1–3). H_2_O_2_ directly oxidizes proteins (Herriott, 1947), but is thermodynamically unstable, decomposing into O_2_ and H_2_O (Pardieck et al., 1992). In the presence of carbonate, H_2_O_2_ became less effective at oxidizing organic compounds, unlike NaOCl (Anderson, 1963). As previously reported (Sykes et al., 1995), NaOCl significantly reduced, but did not completely remove, amino acids in the mollusc shells. We infer that this rapid decrease in amino acid concentration reflects the removal of easily accessed, inter-crystalline, matrix proteins, and thereby isolates an “intra-crystalline” fraction, whose concentration is stable upon further exposure to oxidizing reagents (Fig. 2). The results from this study provide further evidence for a residual fraction of amino acids in molluscs, similar to data published from the bivalve *Mercenaria* (Crenshaw, 1972), and the gastropod *Cepaea* (Sykes et al., 1995). Approximately 10% of the THAA was unaffected by the NaOCl, and is operationally defined as the “intra-crystalline” fraction. In subsequent studies we selected a bleaching procedure that effectively removed inter-crystalline amino acids while minimizing the influence on D/L values; specifically 48 h exposure of a particle size of 0.425–0.09 mm to 12% (w/v available Cl) NaOCl.

Collins and Riley (2000) suggested that isolating the intra-crystalline fraction might eliminate the species differences observed in mollusc shells (e.g., Lajoie et al., 1980), which they believed could be due to differences in the rate of loss of the organic matrix. This was not the case, however. The compositional differences between bleached (intra-crystalline) and unbleached (inter- plus intra-crystalline) shells observed in all three species indicate that the intra-crystalline fraction comprises a different protein fraction than that of the whole shell (Fig. 4). However, a similar compositional shift following bleaching is seen in *Bithynia* and *Margaritifera*, with an increase in the relative abundance of Asx and Glx in the intra-crystalline fraction and a corresponding decline in Ala and Gly. Ala and Gly are common constituents of silk-like proteins (Pereira-Mouries et al., 2002) and these are enriched in the inter-crystalline fraction. The higher relative proportions of Asx and Glx indicates that the intra-crystalline protein within the *Margaritifera* and *Bithynia* shells is more acidic. These findings are consistent with recent investigations on the distribution of silk and acidic proteins within molluscs (e.g., Pereira-Mouries et al., 2002; Falini et al., 2003; Marin and Luquet, 2005). *Margaritifera* has a more primitive shell structure, with a nacreous layer rich in the silk-like proteins. The compositions of the bleached and unbleached *Corbicula* samples are relatively similar, with the Asx-rich protein decreasing in significance in the intra-crystalline fraction (Fig. 4). This suggests that the predominantly crossed-lamellar intra-crystalline fraction of this taxon does not have such a strong division between matrix and entrapped proteins, unlike the prism/nacre of *Margaritifera*.

**Fig. 6:**
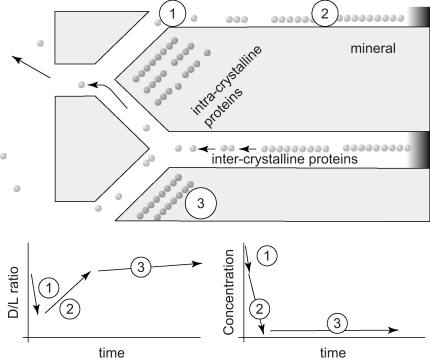
Conceptual model of the effect of bleach on different amino acid fractions in a shell (upper figure modified from Sykes et al., 1995). The highly racemized FAA in the matrix (1) are removed first, leading to a small drop in concentration but a decrease in the D/L value. A rise in D/L coincides with a rapid drop in concentration as the intact proteins of the matrix (2) are removed. Prolonged bleaching may selectively remove amino acids in the intra-crystalline fraction or begins to etch the carbonate, thereby exposing intra-crystalline amino acids (3). This coincides with only a small drop in concentration. At this point the bleaching could induce further racemization, or it could oxidize the less-racemized molecules. The increase in racemization is minimal, however.

The initial slight decrease in D/L value upon bleaching (not observed in some of the experiments) can be explained by the removal of the more highly racemized (and mobile) free amino acids within the inter-crystalline matrix. With increased exposure, the more robust matrix protein is oxidized (predominantly bound amino acids initially less amenable to bleaching than the low molecular weight fraction). The inter-crystalline pool retains relatively low levels of free (and hence more highly racemized) amino acids, the remainder presumably diffusing out of the shell; this fraction *is* retained in the intra-crystalline pool therefore explaining its higher D/L values. The removal of the matrix proteins leaves the more highly racemized intra-crystalline fraction. Upon more prolonged exposure, D/L slowly increases, without a corresponding decline in concentration, suggesting that intra-crystalline amino acids are racemized under the conditions used. The continued increase in D/L implies that the intra-crystalline amino acids are not entirely isolated from the bleach, possibly gaining access via small pores, or causing gradual dissolution of the mineral, releasing d-amino acids. This loss of intra-crystalline amino acids is consistent with the “foam like” structure of the organic matter associated with nanograin crystallites (e.g., Cuif and Dauphin, 2005; Bourrat et al., 2005); these images suggest that the association of organic matter with the mineral phase is intimate, but that the former is not totally enclosed. A schematic of the proposed mechanism is shown in Fig. 6.

## 4 Testing the intra-crystalline fraction using heating experiments

Racemization is slow at room temperature, therefore in order to simulate long-term diagenesis, samples are heated (Hare and Mitterer, 1969). In most experiments, shells are heated in moist sand (e.g., Hare, 1969; but see Brooks et al., 1990). Although this experimental design probably reduces the effect of leaching compared to the heating in water alone, any amino acids that leach out of the shell are lost into the sand. To use racemization data for kinetic models, all the amino acids in the system need to be accounted for (Collins and Riley, 2000). The loss of amino acids by leaching (or other diffusive loss processes) hampers the interpretation of the observed pattern of racemization. In this study, shells were heated in water, which was also analysed to account for all amino acids and to test the integrity of the intra-crystalline fraction.

### 4.1 Heating experiment procedure

Approximately 20 mg of bleached and unbleached (control) powdered *Corbicula*, *Margaritifera* and *Bithynia* shells was weighed accurately into sterile glass ampoules, 300 μL of HiPerSolv water was added, and the glass ampoules sealed. The ampoules were placed in ovens at 110 and 140 °C for varying times. Two to five replicates were removed at specified intervals, up to 2688 h. A sterile plastic-tipped pipette was used to remove 100 μL of the supernatant water for measurement of each the FAA (Fw or FAAw) and the THAA (Hw or THAAw). The aliquots for FAAw were placed in a sterile autosampler vial and dried overnight in a centrifugal evaporator. The aliquots for THAAw were placed in sterile glass vials, with 20 μL/mg equivalent 6 M HCl, flushed with N_2_, then hydrolysed as described above. To quantify the extent of degradation of amino acids in water heated at these temperatures, a solution of free l-amino acids (25 μM of Sigma standard AA-S-18) was heated and analysed in parallel with the shell samples. Shell powders were rinsed six times with HPLC-grade water to remove the supernatant water. The powders were then air-dried overnight and two subsamples were taken for the measurement of the FAA and THAA, prepared as in Section 2.

### 4.2 Heating experiment results

#### 4.2.1 Effect of bleaching pre- versus post-heating

**Fig. 7:**
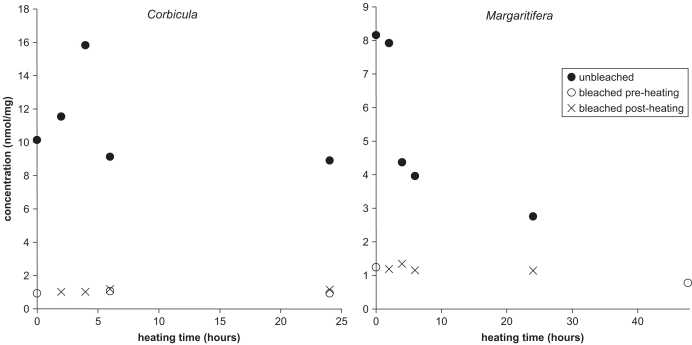
Unbleached, pre-heated bleached, and post-heated bleached concentration of Asx in the THAA under hydrous conditions for *Corbicula* (left) and *Margaritifera* (right) heated at 140 °C.

The focus of this study is on the intra-crystalline fraction, so samples were first bleached to isolate that fraction, then heated to test its behaviour under simulated diagenesis. To evaluate whether the outcome was dependent on whether shells were heated prior to or after bleaching, a subsample of the unbleached powders from the 140 °C bivalve experiments was bleached post-heating (post-heated bleached fraction). The results show that the amino acid concentration is reduced to the same levels irrespective of whether the bleach treatment was conducted before or after simulated diagenesis (Fig. 7). Bleaching pre- and post-heating also resulted in similar D/L values.

#### 4.2.2 Change in amino acid concentration in shell powders on heating

**Fig. 8:**
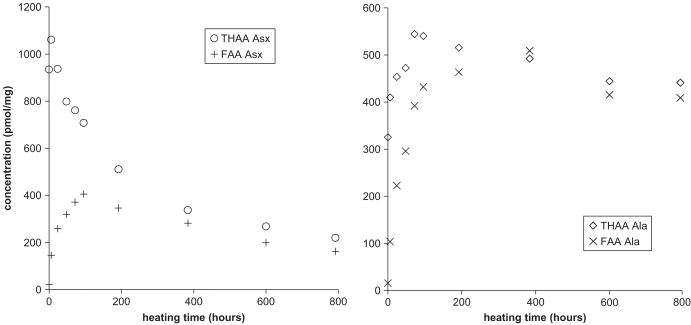
Concentration of Asx (left) and Ala (right) for FAA and THAA (6 h hydrolysis) for the bleached *Corbicula* powder during heating at 140 °C. The increase in abundance of THAA (except Asx) in the early time steps suggests incomplete preparatory hydrolysis.

THAA concentrations in the bleached shells generally decreased with increased heating time, whereas FAA concentrations initially increased, then decreased (Fig. 8). These trends indicate that peptide bonds were hydrolysed during the heating experiments, and that as heating progressed, amino acids were lost to decomposition or leaching. The initial rise in the concentration of most amino acids in the THAA can be attributed to the 6 h preparative hydrolysis procedure, which apparently did not break all of the peptide bonds in these modern shells (cf. Kaufman and Manley, 1998). A longer hydrolysis time of 24 h was used in subsequent analyses.

**Fig. 9:**
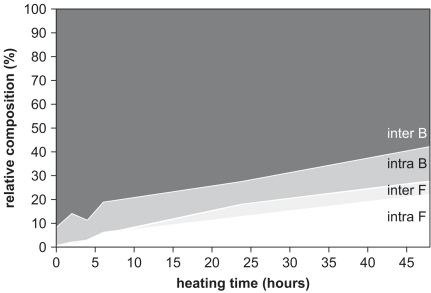
Relative composition of Asx in each of the four fractions of *Bithynia* shell during heating at 140 °C: intra-crystalline free, intra-crystalline bound, inter-crystalline free, and inter-crystalline bound.

By about 200 h at 140 °C, the FAA comprised about 70% of the THAA concentration, and this proportion remained approximately constant with further heating. The difference between the concentration of THAA and FAA with prolonged heating indicates the presence of a residual fraction of bound amino acids. The importance of the intra-crystalline fraction as a percentage of the whole shell increases on heating (Fig. 9). In comparison to the bound amino acids in each fraction, the FAA in the intra-crystalline fraction forms a greater proportion than the inter-crystalline FAA.

#### 4.2.3 Concentration of amino acids in the supernatant water

**Fig. 10:**
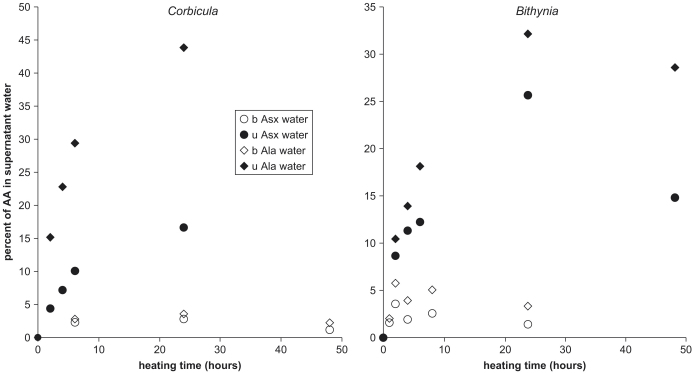
Percentage of THAA in supernatant water (uncorrected for procedural blank) compared to that within the shells, for unbleached and bleached *Corbicula* (left) and *Bithynia* (right) shell at 140 °C. Unbleached shells (u=closed symbols) leach a higher percentage of amino acids into the water compared to bleached shells (b=open symbols).

To quantify the extent that bleached shell (intra-crystalline fraction) approximates a closed system, and to account for all of the amino acids, the supernatant water from the heating experiments was analysed. If the decrease in concentration observed in the heated shells (Fig. 8) was due to leaching, then the leached amino acids would be detectable in the supernatant water. The results show that concentration of amino acids in the supernatant water of the bleached shells was similar to background levels (Table 2). In contrast, the supernatant water of the unbleached powders contained high concentrations of amino acids (Fig. 10). As a proportion of the initial THAA concentration of the shell powders, after 24 h at 140 °C, the THAAw accounts for 1% of the bleached shell compared with 40% of the unbleached shell. Only 20–40% of the amino acids leached from the unbleached shell powders were FAA. Free Glu, however, accounts for only a small proportion of the leached amino acids, presumably due to the formation of its stable lactam (e.g., Walton, 1998). Ala has the highest concentration in the FAAw. This might reflect its relative stability, or its formation as a decompositional product of other amino acids.

The increase in the proportion of Ala is also exhibited by the concentration of amino acids in standard solutions heated under similar conditions. The amino acid concentration in standard solutions decreased by an average of 29% over 24 h at 140 °C (Table 2). The rate of decomposition in the bleached shells is lower than that observed for the FAA solutions, due to the increased stability of peptide-bound amino acids.

### 4.3 Discussion of heating results

Unbleached shells show significant loss (average=40%) of amino acids from the shell into the water upon heating (Fig. 10). In contrast, at both temperatures the paucity of amino acids in the water surrounding the bleached powders (taking into account the decrease due to lower initial concentrations within the powders) demonstrates that the intra-crystalline fraction behaves as a closed system. By comparison, ostrich eggshell heated at 105 °C lost 1% of its stable amino acids to the surrounding water (Brooks et al., 1990) after an initial loss of 10%. The retention of FAA in the intra-crystalline fraction throughout the heating experiments demonstrates the integrity of the system. The decrease in concentration of amino acids in bleach-treated shells on prolonged heating (Fig. 8) can be attributed to amino acid decomposition, a more predictable process that is more easily accounted for in kinetic models than leaching, and that could be a further tool in amino acid geochronology in a closed system.

As the concentration of FAA increased during the initial phases of heating, the THAA concentration decreased (Fig. 8), and because few amino acids were leached out of the intra-crystalline fraction, this loss probably reflects the decomposition of amino acids within the intra-crystalline fraction. The amount of FAA never attained that of the THAA, similar to data on eggshell (Miller et al., 2000), perhaps indicative of a residual fraction of peptide-bound amino acids (e.g., Hoering, 1980). The concentration of THAA might be underestimated because acid hydrolysis causes some decomposition of amino acids (Hill, 1965). Theoretically, all the peptide bonds should eventually be hydrolysed, leaving only FAA. However, refractory material has been reported in a range of studies, from Miocene mollusc shells (Hoering, 1980) to 3.5 Ma fossil brachiopod shells (Walton, 1998) and may explain why very few studies report true equilibrium D/L values of 1.0. This resistant fraction of diagenesis-resistant peptides has never been satisfactorily explained (see discussion in Collins and Riley, 2000). It might reflect a lack of water in the intra-crystalline environment, stable dipeptides, or degradation products with highly resistant or otherwise refractory peptide bonds, such as the stable melanoidin geopolymers, which are only partially released by acid hydrolysis (Abelson and Hare, 1971).

Water is essential for the hydrolysis of the peptide bonds (Hare, 1971) and therefore might be a limiting factor in the protein degradation within a closed system. Shells are estimated to comprise as much as 3% water (Gaffey, 1988), although this is structurally specific and most is as fluid inclusions (Lecuyer and O’Neil, 1993). Additional water could be formed by the decomposition of the hydroxyl amino acids Ser and Thr under mildly alkaline conditions (Towe, 1980; Endo et al., 1995), and by condensation reactions between sugars and proteins (Collins et al., 1992). Nonetheless, the presence of residual bound residues after prolonged diagenesis suggests that water may eventually limit hydrolysis.

#### 4.3.1 Racemization kinetics of the intra-crystalline fraction

**Fig. 11:**
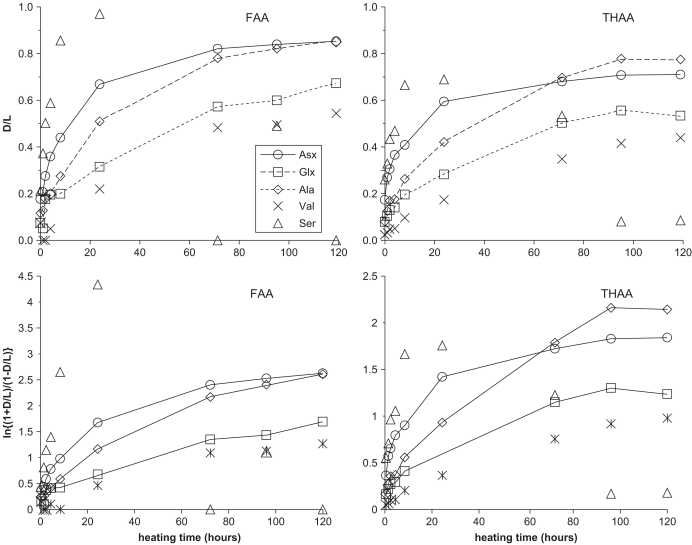
Racemization of bleached *Bithynia tentaculata* shell at 140 °C in the FAA (left) and THAA (right). The upper graphs show the measured D/L values; the lower graphs show the data plotted as ln{(1+D/L)/(1−D/L)} vs. time. If the reaction follows reversible first-order kinetics, the relation should be linear.

All the amino acids within the intra-crystalline fraction showed a systematic increase in the extent of racemization with increased heating time (Fig. 11, upper), except for Ser, which has unusual kinetics. Detailed analysis of the racemization kinetics will be discussed further (Penkman et al., 2007). Like the intra-crystalline fraction of molluscs in this study, ostrich eggshell has been shown to approximate a closed system for the retention of amino acids (Brooks et al., 1990). Isoleucine epimerization in eggshell closely follows first-order reversible kinetics nearly to equilibrium, and this has been ascribed to its closed-system behaviour. In contrast, however, the intra-crystalline fraction in molluscs does not racemize in accordance with first-order kinetics (Fig. 11, lower). Considering the network of reactions that are involved in amino acid racemization in shell proteins, a first-order reaction would not necessarily be expected (Kriausakul and Mitterer, 1978; Wehmiller, 1993). Reversal of trends of D/L vs. time were reported for unbleached mollusc shells heated under similar conditions and time periods (Kimber and Griffin, 1987). This behaviour was not observed in the bleached samples analysed in this study, suggesting that the unusual reversals resulted from leaching of the amino acids from the inter-crystalline matrix of the shells.

#### 4.3.2 D/L values in FAA Versus THAA

In a closed system, the D/L values of amino acids comprising the FAA and the THAA should be highly correlated (Collins and Riley, 2000), because all of the products of diagenesis are retained. In the very early stages of diagenesis the concentrations of FAA are extremely low and so cannot be measured accurately in the small sample sizes used in this study. Throughout most of the range of protein breakdown the FAA D/L is greater than the THAA D/L (Wehmiller and Hare, 1971). At the extreme upper limit of protein degradation both FAA and THAA should display the same DL ratio (1.0) and therefore, given that the slope is less than 1, the relationship is non-linear. Miller et al. (2000), who measured over a wider range of degradation using eggshell samples, observed this non-linear relationship and reported the data as a third order polynomial. However, over the range of data spanned in this study it can be approximated as a linear relationship, with the correlation higher using a linear rather than a polynomial fit (r^2^=0.94). The results show that D/L values of the FAA and THAA are well correlated for each amino acid within the bleached shells, except Ser. Ser racemizes quickly, but rather than slowing towards equilibrium, the (more highly racemized) free Ser is decomposed and the overall extent of racemization in the THAA decreases. Thus, a single D/L value for Ser FAA potentially corresponds to two alternative D/L values for Ser THAA, depending upon the extent of protein degradation (Fig. 11).

**Fig. 12:**
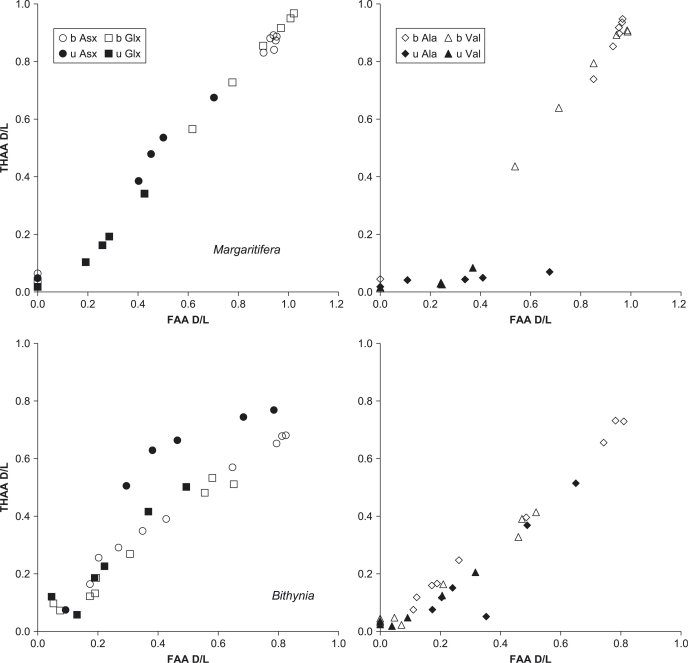
FAA D/L vs. THAA D/L for unbleached (u) and bleached (b) Asx and Glx (left) and Ala and Val (right) for *Margaritifera* (upper) and *Bithynia* (lower) shell heated at 140 °C under hydrous conditions.

The high correlation between D/L values in the FAA and THAA for other amino acids (Fig. 12) indicates that the intra-crystalline fraction retained the highly racemized FAA. The unbleached samples also showed a reasonable correlation between the D/L values for most amino acids; however, the bleached samples tended to have stronger correlations (e.g., Asx and Glx in *Bithynia* shell, Fig. 12, lower left). The extent of racemization of Ala and Val in the THAA of unbleached *Margaritifera* was particularly low (Fig. 12, upper right). This is probably due to the richness of these amino acids in the inter-crystalline matrix and the propensity of the silk-like protein to melt away as (semi-intact) polypeptides (Levi-Kalisman et al., 2001).

## 5 Fossil samples

Fossil shells were analysed to assess the behaviour of intra-crystalline amino acids over Quaternary timescales. Whilst heating experiments on modern samples are useful in mimicking diagenesis in the laboratory, the ultimate aim of our studies is to improve the application of AAR for geochronology. Furthermore, because matrix proteins are easily leached and might be removed early during diagenesis, the utility of the pre-treatment might be diminished for fossil samples.

We focused on two thin-shelled fossil gastropods, *Valvata piscinalis* and *B. tentaculata*, and the thick-shelled bivalve *C. fluminalis*. These species are widely distributed and have been used previously to date UK Quaternary sites as old as 0.5 Ma (Miller et al., 1979; Bowen et al., 1989). Shells were collected from deposits in the Thames Terrace staircase sequence, East Anglia, and other parts of the southern UK (Table 1). Full site details, including independent age information, are given in Penkman et al. (2007). The general preservation of the fossil shells selected for analysis was good, with no evidence of diagenesis, although some post-mortem diagenesis of shell material has been reported from two of the sites: Purfleet (Schreve et al., 2002) and Barnham (Preece and Penkman, 2005). At two sites, Funthams Lane and Purfleet, natural variability among shells was explored further by analysing a large number of samples. For the unbleached/bleached comparison studies, 87 individual *Bithynia*, 58 *Valvata* and 17 *Corbicula* shells were analysed for FAA and THAA. All samples were prepared and analysed as in Section 2. The samples are considered in this study simply as representatives of a fossil population; the geological significance of the results obtained is discussed in further detail elsewhere (Penkman, 2005; Penkman et al., 2007).

### 5.1 Fossil sample results

#### 5.1.1 Samples from multiple stratigraphic horizons

**Fig. 13:**
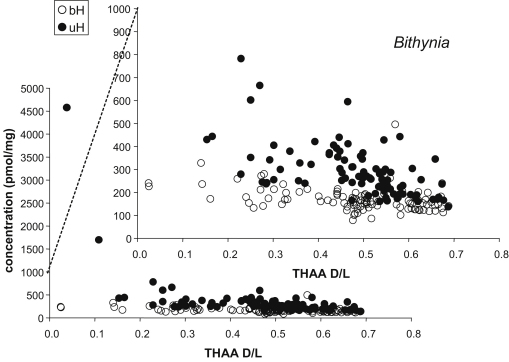

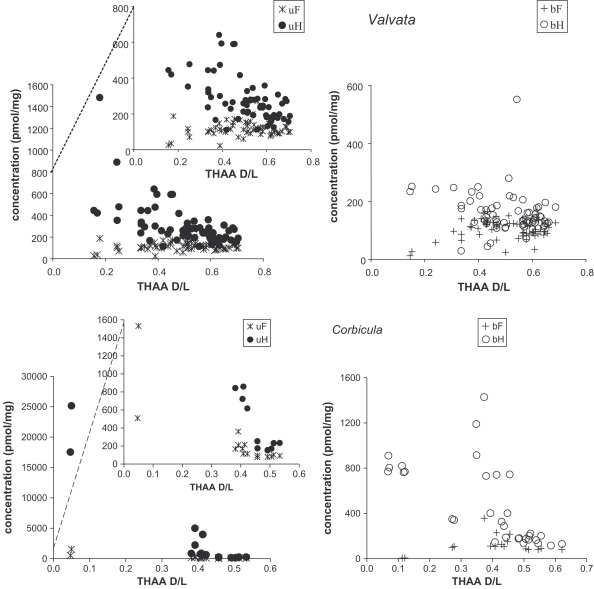
Concentration vs. THAA D/L for Asx for bleached and unbleached fossil shells. Upper: [THAA] for *Bithynia*. Middle: [THAA] and [FAA] for unbleached (left) and bleached (right) *Valvata*. Lower: [THAA] and [FAA] for unbleached (left) and bleached (right) *Corbicula*. Insets show an expanded scale.

The concentration of THAA decreased dramatically with bleaching, especially at low D/L values (Fig. 13). The variability among shells also decreased. All amino acids for all three species show generally similar trends. The concentrations of the unbleached samples tend to be higher than that of the bleached shells, even at high D/L values (Fig. 13). Little change in the concentration or D/L values of the FAA was observed in the bleached shells. For Asx, the THAA concentration generally decreased with increasing D/L, and with a concomitant increase in FAA concentration in both bleached and unbleached shells (Fig. 13); however, the relative proportion of FAA was higher for the bleached samples. For Ala, the intra-crystalline fraction of *Bithynia* was dominated by the FAA for shells with D/L>0.1. The decline in THAA following bleaching tended to be greater for the thick shelled *C. fluminalis*, particularly at high (>0.4) D/L values, than it was for the thin shelled gastropods (Fig. 13).

#### 5.1.2 Multiple shells from single horizons: replicability

Three sets of fossil samples from two Quaternary sites were analysed to test the effect of the bleach treatment on multiple samples from single horizons: 25 *B. tentaculata* and 15 *V. piscinalis* shells from Funthams Lane (correlated with MIS 7; Langford et al., 2007), and 10 *B. tentaculata* shells from Purfleet (correlated with MIS 9; Schreve et al., 2002). The shells from each horizon presumably represent a single age population (at both sites the faunas have ecological integrity, suggesting reworking has been minimal or non-existent; at Purfleet several of the larger bivalves can be seen in life-position with united valves), and provide a test of the replicability of the amino acids within the intra-crystalline fraction.

**Fig. 14:**
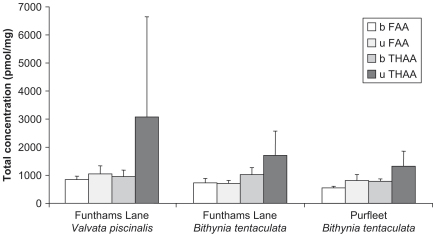
Concentrations for unbleached (u) and bleached (b) FAA and THAA fractions for *Valvata piscinalis* and *Bithynia tentaculata* from Funthams Lane, and *Bithynia tentaculata* from Purfleet. Error bars are one standard deviation about the mean. Little change is observed upon bleaching for the FAA.

**Fig. 15:**
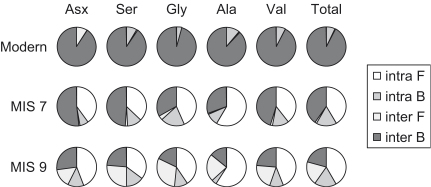
Average amino acid compositions for intra- and inter- free (F) and bound (B) amino acids within modern, MIS 7, and MIS 9 *Bithynia tentaculata*.

**Fig. 16:**
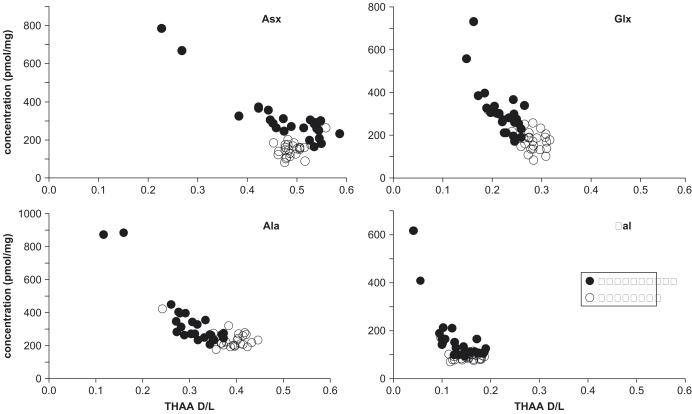
THAA concentration vs. D/L for Asx, Glx, Ala, and Val for bleached and unbleached *Bithynia tentaculata* from Funthams Lane.

**Fig. 17:**
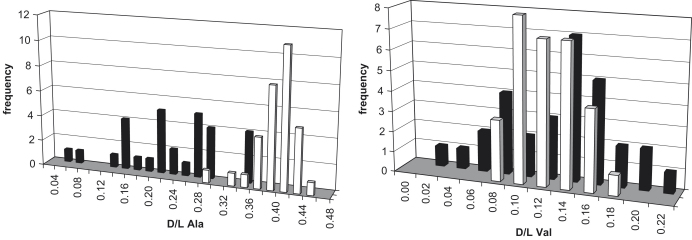
Frequency distribution of THAA D/L for Ala and Val for unbleached (■) and bleached (□) *Valvata piscinalis* from Funthams Lane.

The concentration of FAA did not tend to show a significant difference between bleached and unbleached samples (Figs. 14 and 15). In contrast, bleaching dramatically affected the concentration and D/L of the THAA for all three taxa (Figs. 14 and 16). The concentration of THAA decreased with bleaching, especially at low D/L values (Fig. 16). The bleached samples show much tighter clustering, although one bleached *Bithynia* shell from Funthams Lane is an outlier. The variability in D/L values was also reduced by bleaching, as represented by the reduced size of the “footprint” of the frequency distribution (Fig. 17). In some amino acids, such as Ala in *Valvata* from Funthams Lane, the mean D/L value also increased significantly in the bleached shells (Fig. 17).

**Fig. 18:**
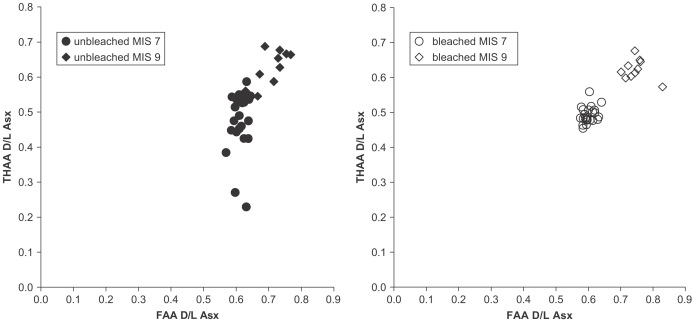
THAA vs. FAA D/L Asx for bleached *Bithynia* shells from Funthams Lane (MIS 7) and Purfleet (MIS 9). Each of the measurements is from a single shell. Note the much greater consistency in the measurements obtained from bleached shells.

Plotting the extent of racemization of THAA against FAA presents the samples in relative aminostratigraphical order based on their D/L values, with more degraded samples having higher values of D/L for both the FAA and THAA fractions. Separation between the MIS 7 Funthams Lane *B. tentaculata* shell and the MIS 9 *Bithynia* shell is better upon bleaching (Fig. 18).

The protein composition of shell changes as it ages (Fig. 15). The increase in the importance of the FAA with age indicates its generation by protein hydrolysis. The intra-crystalline fraction becomes more dominant with age, which supports the hypothesis that matrix protein is progressively leached from fossil shells. However, the degree of leaching is specific to the depositional environment: the samples from Purfleet, although older than those from Funthams Lane, contain a larger proportion of inter-crystalline FAA.

**Fig. 19:**
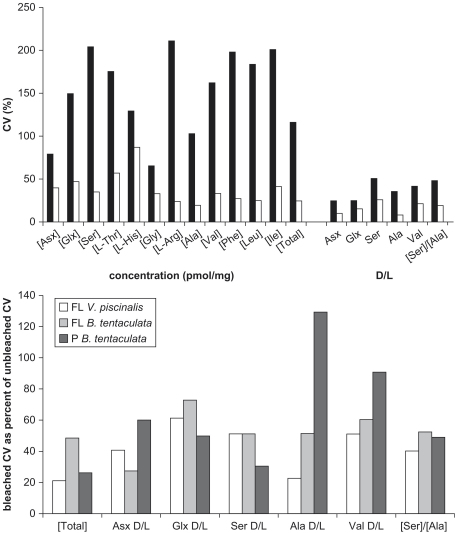
Coefficient of variation (CV) of the concentrations and D/L values of THAA in unbleached and bleached fossil samples. Upper: 15 individual unbleached (■) and bleached (□) *Valvata piscinalis* shell powders from Funthams Lane. Lower: CVs of bleached samples as a percentage of the CVs for unbleached samples, for the THAA concentration and D/L values from Funthams Lane (FL) *Valvata piscinalis*, *Bithynia tentaculata* and from Purfleet (P) *Bithynia tentaculata*. Inter-shell variability is reduced in all measures of the amino acid composition, except for D/L Ala in shells from Purfleet, where the CVs are low.

The coefficient of variance $\left(\mathrm{CV}=\bar{x}/\sigma \times 100\%\right)$, also called the relative standard deviation, is a measure of variability that takes into account the magnitude of the mean. The CV can be used to assess the inter-shell variability of amino acid concentrations and D/L values while avoiding problems posed by the change in magnitude of values caused by the bleach treatment (Fig. 19). In all but two of the 57 measurements made across both species at the two sites, the CV decreased for the bleached samples. In those two measurements (the D/L Ala for *B. tentaculata* at Purfleet, and [Glx] in *B. tentaculata* at Funthams Lane) the CVs for both the bleached and unbleached samples were low. On average, the CV for D/L values decreased by 51% in the intra-crystalline fraction, which is indicative of the improvement that results from bleaching (Fig. 19, lower). The CVs of the concentration data are much higher than for the D/L values. This is likely to reflect the inherent inaccuracies in calculating the concentration of amino acids within a sample, which is dependent on the accuracy of the measurements of the small masses (mg) of samples and volumes (μL) of spiked reagents.

The CVs for the D/L values in the intra-crystalline fraction average 10% for Asx, Glx, Ala, and Val. Although lower than for the unbleached samples, this is not as low as previously suggested for single-age population. The inter-shell variability for a well-behaved sample of bivalves is typically 6–8% for aIle/Ile (based on peak-height values) (Miller and Brigham-Grette, 1989), and 3–5% for aIle/Ile in ostrich eggshells (Miller et al., 1992). The high CVs, even within the intra-crystalline fraction, may be due to higher levels of natural variability within gastropods; thick-shelled marine bivalves have given more reproducible results than gastropods in Quaternary marine terrace deposits (Lajoie et al., 1980). In contrast, McCoy (1987) reports that a reasonable standard error for the mean A/I in a single genus of molluscs is 5%; the standard error on the bleached gastropods in our single-horizon analyses averages 1%.

#### 5.1.3 Inter-laboratory comparison samples

**Fig. 20:**
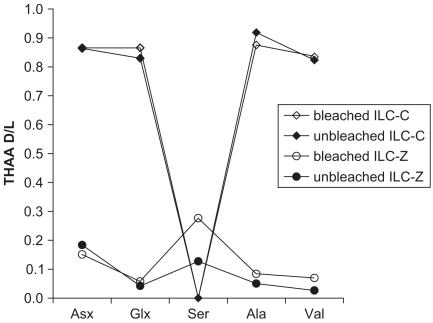
THAA D/L for Inter-laboratory comparison samples C (ILC-C) and Z (ILC-Z), unbleached and bleached.

The inter-laboratory comparison samples (ILC; Wehmiller, 1984; Kaufman and Manley, 1998) span a larger time period and range of D/L values than represented within the UK fossil material analysed in this study, therefore providing a further test of the bleaching protocol. Bleached THAA samples tend to yield higher D/L values, but the differences diminish with age (Fig. 20). Little difference is observed between bleached and unbleached samples in the FAA fraction. Similar results have been obtained on bleached and unbleached ILC samples analysed by gas chromatography (J. F. Wehmiller, pers. comm.).

### 5.2 Fossil samples discussion

For the fossil gastropods, the greatest difference in amino acid concentration between the unbleached and bleached shells tends to be in the younger samples (low D/L values; Figs. 13 and 20). Amino acids probably leach out of the thin-shelled gastropods relatively early in the diagenetic process, much like that observed in ratite eggshell (Miller et al., 2000), resulting in a higher proportion of intra-crystalline amino acids with increasing age. However, the abundance of inter-crystalline amino acids that survive during burial appears to vary among the samples analysed. Whilst similar bleaching experiments performed on the thin-shelled Pleistocene gastropod, *Lymnaea*, showed little change in composition, indicating that the amino acids in the inter-crystalline matrix of these shells may have been removed during burial (Kaufman, unpublished), the results of our single-horizon study show that shells even in the same depositional environment have variable compositions of inter-crystalline amino acids. Shells with a higher portion of the inter-crystalline amino acids exhibit lower D/L values in the THAA fraction. This trend may represent partial leaching of the inter-crystalline matrix of proteins, with some of the unbleached samples having lost all their inter-crystalline protein even before bleaching. A similar trend would also be observed if the samples had been contaminated with younger amino acids, indicating that treatment with bleach has two advantages: removal of the leachable matrix proteins and removal of contaminating material.

One of the bleached *Bithynia* shells from Funthams Lane exhibits a higher concentration and lower D/L for Glx, Ala, and Val compared with the others. This outlier plots within the trend defined by the unbleached shells, indicating that this shell was not bleached fully.

The observed increase in FAA concentration with increasing D/L is expected, due to the hydrolysis of the peptide bonds. Bleaching did not significantly affect the concentration and composition of the FAA (Figs. 13 and 14). The inter-crystalline fraction removed by the bleach contains very low concentrations of FAA. The FAA within the fossils dominantly resides in the intra-crystalline fraction. A closed system is more likely to retain the FAA, whereas in the unbleached open system these small molecules are easily leached from the inter-crystalline fraction into the burial environment, representing a lower proportion of the THAA in the unbleached fraction. The proportion of free Glx is noticeably lower than that for other amino acids, reaching only 50% free in even the oldest samples. This is attributed to lactam (pyroglutamic acid) formation from free Glx; this imine is not detected under the analytical conditions used. The concentration of free Glx therefore underestimates the actual abundance of Glx released by hydrolysis.

#### 5.2.1 Correlation between D/L values in the FAA and THAA

**Fig. 21:**
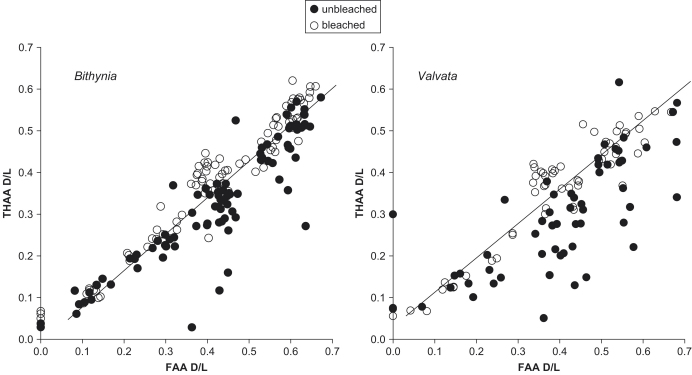
THAA D/L vs. FAA D/L for Ala for bleached (●) and unbleached (○) *Bithynia* (left) and *Valvata* (right). The trendlines are derived from kinetic experiments on the intra-crystalline fraction of the two species.

**Fig. 22:**
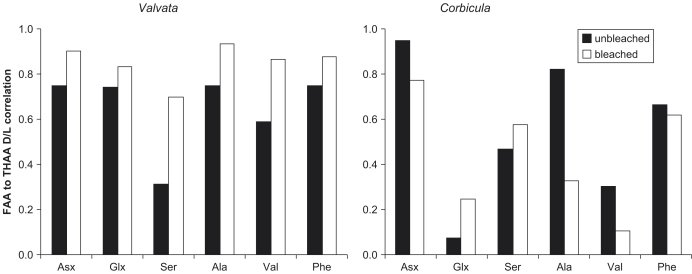
FAA to THAA D/L correlation for each amino acid for bleached and unbleached *Valvata* (left) and *Corbicula* (right). There is no improvement upon bleaching for *Corbicula*, unlike that observed for the fossil gastropods.

In a closed system, the relation between the extent of racemization in the FAA and THAA should be predictable. The results of this study show that the correlation is significantly improved following bleaching for both gastropod species (Figs. 21 and 22). This high correlation, similar to that derived from the high-temperature experiments, indicates that there is no preferential loss of free amino acids within the fossil shells and demonstrates the integrity of the intra-crystalline fraction, approximating closed system behaviour (Miller et al., 2000). For the fossil *Bithynia* (figure not shown), the correlation between the D/L values is improved for Asx, Ser, Ala, Val, and Phe but is slightly lower for Glx, although both unbleached (0.90) and bleached (0.88) values in this case are high. The correlation between FAA and THAA D/L values for Ser tends to be low, likely to be due to its complicated racemization/decomposition kinetics.

Nearly all of the points that fall off the trend defined by the others have unexpectedly low THAA D/L relative to FAA D/L values. These outliers fall into two groups: those with higher concentrations, likely to have been contaminated by exogenous amino acids; and those with lower concentrations, hypothesized to have lost their highly racemized FAA and short peptides from the inter-crystalline fraction, leaving the less-leachable, long-chain protein with lower D/L values. This pattern is also consistent with the idea that the majority of the FAA in the unbleached samples is contained with the intra-crystalline fraction.

#### 5.2.2 Corbicula

The clear improvement in the correlation between D/L values in the FAA and THAA that was observed for the gastropods was not seen in *Corbicula*. The correlation increased for Glx, but bleaching resulted in decreased correlations for the other amino acids (Fig. 22). The difference could be due to incomplete bleaching, although the amino acid concentration decreased in the same way on bleaching *Corbicula* as it did for the gastropod shells. Alternatively, the difference could reflect differing protein composition or shell structure. The cross-lamellar structure of *Corbicula* might be more susceptible to incomplete bleaching than the thin-shelled gastropods. *Corbicula* accretes different structural layers dependent on its environment, which may account for the inconsistencies in the amino acids (Tan and Prezant, 1989). Indiscriminate sampling of the shells might have resulted in an uneven mixture of these layers; with different kinetic patterns, this would result in a confused dataset. Furthermore, the proteins in *Corbicula* are enriched in Asx compared to the gastropods. Asx forms the most-easily hydrolysable peptide bond, which might consume available water thereby reducing the rate of hydrolysis involving other amino acids. However, the results from fossils and the kinetic experiments do not indicate that Asx is released significantly faster than other residues.

Problems with AAR geochronology using the bivalve *Corbicula* have previously been reported (Miller et al., 1979; Bowen et al., 1995). At the Purfleet site, *Corbicula* exhibited high inter-shell variability and high D/L values, inconsistent with the proposed age of the site. Bowen et al. (1995) had previously attempted bleaching on these shells, without improving the dataset. The bleaching experiments carried out on heated modern *Corbicula* in this study did successfully isolate a residual intra-crystalline fraction of amino acids that did not leach, but bleaching of fossil *Corbicula* did not provide a more consistent protein fraction.

#### 5.2.3 Covariance of D/L in Asx and Glx for single horizon

**Fig. 23:**
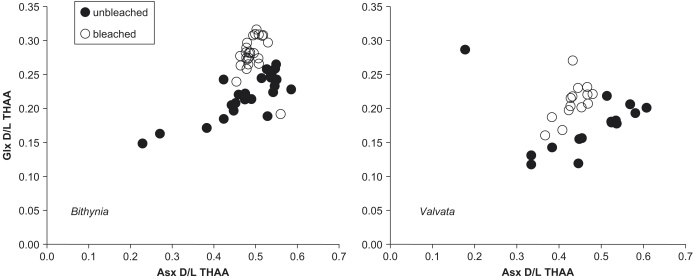
THAA D/L for Glx vs. Asx for Funthams Lane *Bithynia tentaculata* (left) and *Valvata piscinalis* (right). The results on bleached shells form a more coherent cluster, except for one outlier, which contains unexpectedly high amino acid concentration and is probably incompletely bleached.

The well-defined covariance of D/L Glx and D/L Asx has been used previously for objectively identifying and excluding aberrant results (e.g., Kaufman, 2006). The D/L values from bleached *B. tentaculata* and *V. piscinalis* from single horizons cluster more closely than do their unbleached counterparts (Fig. 23). The one outlier might reflect incomplete bleaching. Such plots can be useful for identifying outliers in bleached shells, although bleaching reduces or eliminates the need for data screening, potentially a major advantage of the procedure.

## 6 Summary and conclusions

**Fig. 24:**
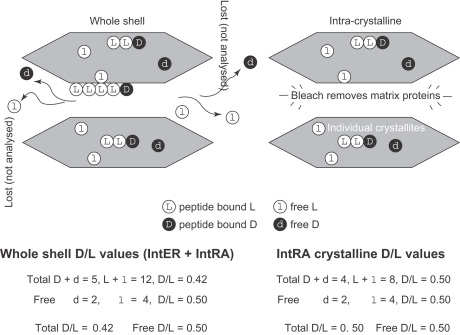
Schematic of intra-crystalline amino acids entrapped within carbonate crystals. Unlike the proteins of the organic matrix between the crystallites, which leach from the shell with time, amino acids are entrapped in the closed intra-crystalline system. Thus the relation between the D/L values of different amino acids and between free (non-protein bound) and total hydrolysable (both free and originally protein-bound amino acids, released by acid hydrolysis) amino acids is predictable. Analysis of the whole shell results in lower-than-expected D/L values for the THAA, due to the loss of the more highly racemized FAA.

Exposure of powered mollusc shells to concentrated NaOCl for 48 h effectively reduces the amino acid content of the three mollusc taxa in this study to residual levels. We infer that this procedure removes the readily accessed organic matrix that resides between mineral crystals while isolating the intra-crystalline fraction. Fig. 24 illustrates schematically the influence of bleaching by comparing the concentration and extent of racemization in amino acids before (whole shell) and after (intra-crystalline) bleaching. The model accounts for the higher percent FAA and higher D/L values in the intra-crystalline fraction. The lack of FAA in the inter-crystalline fraction indicates that these have been lost through diagenesis, and as these tend to be more highly racemized than the THAA fraction (Mitterer and Kriausakul, 1984), this loss would decrease the D/L for the THAA of the whole shell. Whilst the proportion of intra-crystalline amino acids within the whole shell becomes increasingly important as the sample ages, the abundance of amino acids in the inter-crystalline matrix is variable, even for shells within the same depositional environment.

The results of this study show that the intra-crystalline fraction of mollusc shells approximates a closed system for the retention of amino acids. When heated at 140 °C for 24 h, only 1% of the total amino acid content of *Bithynia* shell is leached from the intra-crystalline fraction into the surrounding water. This compares with 40% of amino acid content that is leached from unbleached shells under the same conditions. This loss of amino acids from the intra-crystalline fraction is similar to that of ostrich eggshell (Brooks et al., 1990). The closed-system behaviour of the intra-crystalline fraction is also indicated by the strongly correlated D/L values in the FAA and THAA populations of bleached shells (average *r*^2^=0.90 for Asx, Glx, Ala, Val, and Phe). Finally, the concentration of the FAA is little affected by bleaching, indicating that these amino acids are effectively retained within mineral crystals, despite their greater susceptibility to leaching compared with bound amino acids. The rate of racemization in the intra-crystalline fraction of shells heated isothermally does not conform to first-order reversible kinetics. This can be explained by the influence of hydrolysis on the rate of racemization, which differs depending on the structural position of amino acids (Kriausakul and Mitterer, 1980).

The composition of the intra-crystalline fraction in modern *Bithynia* and *Margaritifera* is enriched in acidic amino acids compared with the inter-crystalline amino acids, with the relative proportion of Asx and Glx about 10% higher. The composition of the intra-crystalline fraction differs among the three taxa, even for related genera (*Bithynia* and *Valvata*; Gastropoda, Prosobranchia). Furthermore, the extent of racemization in the intra-crystalline fraction differs for different species from the same site and presumably of the same age. The intra-crystalline fraction exhibits taxon-dependent differences similar to conventionally prepared shells.

The results of this study show that isolating the intra-crystalline fraction reduces the variability among fossil gastropod shells, one of the major sources of uncertainty for amino acid geochronology (Murray-Wallace, 1995). The inter-shell variability for amino acid concentration and racemization are reduced by 68% and 49%, respectively, in the intra-crystalline fraction of a single-age population compared with conventionally prepared, unbleached shells. The reduced variability exhibited by the intra-crystalline fraction can be attributed to its closed-system behaviour. Amino acids within crystals are effectively isolated from variable external rate-affecting factors, including contamination by exogenous amino acids, microbial decomposition, and leaching. In contrast, inter-crystalline amino acids undergo a wider range of diagenetic pathways, leading to variation in the concentration and D/L. The covariance of the extent of racemization in the FAA and THAA populations can be used to assess the integrity of the closed system, enabling the identification of compromised samples. The improvement in the precision of geochronological interpretations afforded by the intra-crystalline fraction is commensurate with its reduced variability.

## Figures and Tables

**Table 1:** Samples analysed in this study

Species	Location	Age	*n*^a^	Individual or bulked samples	Oxidized H_2_O_2_ (h)	Oxidized NaOCl (h)	Heated 110 °C (h)	Heated 140 °C (h)
*Corbicula fluminalis*	Snake River, Idaho	Holocene	4	B	0–264	0–240	0–2688	0–792
*Margaritifera falcata*	Snake River, Idaho	Modern	3	B	0–264	0–120	0–1344	0–864
*Bithynia tentaculata*	Stratford-upon-Avon	Holocene	50	B				0–96
*Bithynia tentaculata*	Cambridge	Modern	10	B		0–120		
*Valvata piscinalis*	Funthams Lane, Unit G3	Pleistocene	15	I		48		
*Bithynia tentaculata*	Funthams Lane, Unit G3	Pleistocene	25	I		48		
*Bithynia tentaculata*	Purfleet, sample 6	Pleistocene	10	I		48		
*Bithynia tentaculata*	Samples from 18 sites	Pleistocene	53	I		48		
*Valvata piscinalis*	Samples from 12 sites	Pleistocene	43	I		48		
*Corbicula fluminalis*	Samples from 6 sites	Pleistocene	17	I		48		

Full site details are given in Penkman et al., 2007.

a number of individual shells analysed.

**Table 2:** Average ‘Total’ concentrations of *Bithynia tentaculata* shell and the supernatant water after 24 h of heating at 140 °C

Sample	Initial [Total]^a^ THAA (pmol/mg)	[Total] THAA in shell after 24 h @ 140 °C (pmol/mg)	[Total] THAAw in water after 24 h @ 140 °C (pmol/mg equiv)^b^	Overall loss in shell (%)	Loss into water (%)	Loss by decomposition (%)^c^
Unbleached *Bithynia*	25037±6680 (*n*=6)	5117±738 (*n*=2)	10096±2861 (*n*=2)	80	40	39
Bleached *Bithynia*	1391±23 (*n*=4)	1304±34 (*n*=2)	10±64 (*n*=2)	6	0.70	5
FAA standards	110±0.3 (*n*=2)		78 (*n*=1)			29

a Total concentration [total]=concentration of Asx, Glx, Ser, Gly, Ala, Val, and Phe. The procedural water blank contained 56 pmol/mg equiv [total] THAA after 24 h of heating. This value is subtracted from the calculated THAAw [total] in this table.

b pmol/mg equiv=100 μl of supernatant water was analysed for THAAw, out of an original volume of 300 μl. Concentrations were calculated as the fraction of the original shell mass represented by this volume of water.

c % decomposition=The percentage of the original amino acid concentration lost from the shell, but not leached into the water, therefore assumed to have decomposed.
